# Analysis of Bioavailability and Induction of Glutathione Peroxidase by Dietary Nanoelemental, Organic and Inorganic Selenium

**DOI:** 10.3390/nu13041073

**Published:** 2021-03-25

**Authors:** Mitchell T. Ringuet, Billie Hunne, Markus Lenz, David M. Bravo, John B. Furness

**Affiliations:** 1 Department of Anatomy & Neuroscience, University of Melbourne, Parkville, VIC 3010, Australia; mringuet@student.unimelb.edu.au (M.T.R.); b.hunne@unimelb.edu.au (B.H.); 2 Florey Institute of Neuroscience and Mental Health, Parkville, VIC 3010, Australia; 3 Institute for Ecopreneurship, School of Life Sciences, University of Applied Sciences and Arts Northwestern Switzerland, 4132 Muttenz, Switzerland; markus.lenz@fhnw.ch; 4 Sub-Department of Environmental Technology, Wageningen University, 6700 AA Wageningen, The Netherlands; 5 Innovation and Technology, Land O’Lakes Inc., Arden Hills, MN 55112, USA; DBravo@landolakes.com; 6 Department of Agriculture & Food, University of Melbourne, Parkville, VIC 3010, Australia

**Keywords:** selenium, bioavailability, nanoelemental selenium, animal feed, antioxidant enzymes

## Abstract

Dietary organic selenium (Se) is commonly utilized to increase formation of selenoproteins, including the major antioxidant protein, glutathione peroxidase (GPx). Inorganic Se salts, such as sodium selenite, are also incorporated into selenoproteins, and there is evidence that nanoelemental Se added to the diet may also be effective. We conducted two trials, the first investigated inorganic Se (selenite), organic Se (L-selenomethionine) and nanoelemental Se, in conventional mice. Their bioavailability and effectiveness to increase GPx activity were examined. The second trial focused on determining the mechanism by which dietary Se is incorporated into tissue, utilising both conventional and germ-free (GF) mice. Mice were fed a diet with minimal Se, 0.018 parts per million (ppm), and diets with Se supplementation, to achieve 0.07, 0.15, 0.3 and 1.7 ppm Se, for 5 weeks (first trial). Mass spectrometry, Western blotting and enzymatic assays were used to investigate bioavailability, protein levels and GPx activity in fresh frozen tissue (liver, ileum, plasma, muscle and feces) from the Se fed animals. Inorganic, organic and nanoelemental Se were all effectively incorporated into tissues. The high Se diet (1.7 ppm) resulted in the highest Se levels in all tissues and plasma, independent of the Se source. Interestingly, despite being ~11 to ~25 times less concentrated than the high Se, the lower Se diets (0.07; 0.15) resulted in comparably high Se levels in liver, ileum and plasma for all Se sources. GPx protein levels and enzyme activity were significantly increased by each diet, relative to control. We hypothesised that bacteria may be a vector for the conversion of nanoelemental Se, perhaps in exchange for S in sulphate metabolising bacteria. We therefore investigated Se incorporation from low sulphate diets and in GF mice. All forms of selenium were bioavailable and similarly significantly increased the antioxidant capability of GPx in the intestine and liver of GF mice and mice with sulphate free diets. Se from nanoelemental Se resulted in similar tissue levels to inorganic and organic sources in germ free mice. Thus, endogenous mechanisms, not dependent on bacteria, reduce nanoelemental Se to the metabolite selenide that is then converted to selenophosphate, synthesised to selenocysteine, and incorporated into selenoproteins. In particular, the similar efficacy of nanoelemental Se in comparison to organic Se in both trials is important in the view of the currently limited cheap sources of Se.

## 1. Introduction

Oxidative stress, which is a common occurrence in livestock that can be caused by a wide range of factors, compromises gut health and reduces the efficiency with which the intestine absorbs nutrients [1,2]. This intestinal dysfunction can be ameliorated by increasing antioxidant activity, for example through administration of selenium (Se) or vitamin E [2,3]. Se is incorporated into selenocysteine (SeCys), which can then form part of selenoenzymes, including glutathione peroxidase (GPx) [4]. The bioavailability of Se regulates GPx activity, supplemental Se generally increasing its total catalytic activity. For this reason, Se is added to animal food to reduce oxidative stress [5]. The least expensive form is inorganic Se salts, usually sodium selenite, but inorganic Se incorporation into tissues varies [6]. Organic Se, in the form of L-selenomethionine (SeMet), is more efficiently incorporated, but its production process makes it more expensive. SeMet can substitute for methionine although it has been reported to have adverse effects at high doses [7,8,9]. Following ingestion, dietary SeMet and sodium selenite are both reduced to hydrogen selenide (H_2_Se) that is a substrate for SeCys that is incorporated into selenoproteins [4,6]. SeMet can be trans-selenated to SeCys, which is then converted to H_2_Se by SeCys β-lyase [4]. On the other hand, sodium selenite reduces to H_2_Se through interaction with glutathione (GSH), producing selenodiglutathione (GSSeSG), which is further metabolised by GSH reductase into glutathioselenol (GSSeH), GSSeH either decomposes to GSH and Se^0^ or is converted to H_2_Se through enzymatic and non-enzymatic pathways [10]. H_2_Se is then converted to selenophosphate (HSePO_3_^2−^) for downstream incorporation into selenoproteins such as GPx, or excretion in urine as a selenosugar [9]. Another cheap, less toxic Se form, nanoelemental Se, has shown promising results to reduce antibiotic use in livestock [11], but its mechanism of action in eukaryotic systems is not understood.

The production of nanoelemental forms of Se can be achieved by biological reduction in the oxyanions, selenite and selenite, or by chemical means [12,13,14]. Elemental Se in amorphous (i.e., non X-ray diffracting) form (often referred to as “nanoelemental red Se”) could be a cheaper source of dietary selenium if its in vivo conversion is efficient. Recent evidence suggests that nanoelemental Se in the diet could be utilized as a selenoprotein source [15,16]. However, it is not clear how effectively nanoelemental Se can be solubilized, taken up from the diet, and ultimately incorporated in reduced form into selenoproteins, and whether this depends on intestinal bacteria. Elemental Se oxidation mechanisms in bacteria have been proposed, therefore we tested the hypothesis that the absorption and utilization of nanoelemental Se in mammals could be due to bacterial action [17,18,19]. Bacteria can oxidize elemental Se to Se^+VI^, selenate [19] and sulphate reducing bacteria [20] could feasibly also reduce selenate and selenite to H_2_Se, that could be readily incorporated into selenoproteins [21]. The purpose of these experiments is to compare the bioavailability of nanoelemental Se with other Se forms and to investigate mechanistically whether its absorption is dependent on bacteria.

## 2. Materials and Methods

The study utilized C57Bl/6 mice from the Animal Resources Centre (ARC) (Perth, Australia) and the Walter and Eliza Hall Institute (WEHI, Melbourne, Australia) germfree mouse facility (Melbourne, Australia). Ethics was approved by the University of Melbourne and Florey Institute Animal Ethics Committees.

The Se formulations that were investigated were sodium selenite (Na_2_SeO_3_, Sigma, Sydney, Australia), l-selenomethionine (SeMet; Sigma) and nanoelemental Se, as pure red amorphous elemental Se nanoparticles in an aqueous suspension. Nanoelemental Se was produced in the laboratory by reducing selenite (Se^+IV^) with reduced glutathione (GSH) in the presence of bovine serum albumin (BSA). For this, 2.4 mL of 25 mM sodium selenite in water was added to 9.6 mL water containing 73.8 mg reduced glutathione and 480 mg BSA. Sodium hydroxide (1M) was added dropwise until red coloration appeared. After this reaction, the nanoparticles were cleaned from residues of the reaction by repeated (5×) ultrafiltration (65,000 rcf, 1 h) and washing with nanopure water. After several washing steps, ultrafiltration and LC-ICP-MS was used to ensure as previously described to ensure that no residual selenite was present. A small aliquot (2–3 µL) of the washed suspension was dried on a silicon wafer before sputtering using Au−Pd. Particles sizes were then determined by scanning electron microscopy (SEM) on a Supra 40 V system (Carl Zeiss, Hombrechtikon, Switzerland) using an in-lens and a secondary electron detector as previously described [22]. The Se nanoparticle yielded had an average diameter of 120 nm. The concentration of the final Se nanoparticle suspension was quantified by ICP-MS after aqua regia digestion.

Selenium levels in prepared foods were determined by mass spectrometry using a Gas Chromatography Mass Spectrometer (GC-MS) at the National Measurement Institute (Melbourne, Australia).

### 2.1. Bioavailability Mouse Experiments

Weaned C57Bl/6 mice, 4 weeks old, were placed on the prepared diets detailed below (0.018 ppm Se in unsupplemented food, and total Se 0.07, 0.15 and 1.7 ppm in the supplemented food). Concentrations were chosen based on the current Food and Drug Administration (FDA) requirements of complete feed for livestock to not exceed 0.3 ppm (FDA: Sec. 573.920, section (c) (1), 2019), with two diets below required threshold and one well above, at 1.7 ppm, added to determine if excessive inclusion would increase GPx activity or result in detectable toxicity. The source Se was made up to the desired concentration in distilled water and was added fresh each day to powdered Speciality Feeds low Se (0.018 ppm Se) diet (Speciality Feeds, Perth, Australia) and mixed thoroughly. The low Se diet (SF16-014) was based on the standard AIN (American Institute of Nutrition) 93 rodent diet, but with no added selenium and with 30 g/100 g Torula yeast used instead of casein as the protein source to further reduce selenium. Extra vitamins were added to diets to account for expected losses during irradiation. The complete list of ingredients added to the diet can be found below (Table 1). Diets were irradiated at 25 kGy (Steritech irradiation, Melbourne, Australia). The experiment was a 3 × 3 factorial design with levels (0.07, 0.15, and 1.7 ppm) and sources (inorganic, organic and nanoelemental Se) as the independent variables (6 mice per treatment; 3 males and 3 females). An additional treatment (control; 0.018 ppm Se) was added (*n* = 14).

### 2.2. Inorganic Sulphur Exclusion Experiments

For sulphate exclusion experiments, the base AIN93 rodent diet contained no added Se or sulphate, in the form of K_2_ SO_4_. Total Se present for the diets (organic, inorganic or nanoelemental Se) was 0.3 ppm. Total sulphur (S) present was 5600 ppm (5.6 mg/kg), primarily derived from sulphur-containing amino acids (cysteine, methionine). Mice were placed on the organic sulphur diets (5600 ppm) with or without potassium sulphate (K_2_SO_4_) added to yield 0.6 g inorganic sulphur/kg, i.e., 600 ppm S. Se formulations were mixed with the organic S or the K_2_SO_4_ supplemented diet and irradiated at 25 kGy (Steritech irradiation, Melbourne, Australia). Groups of eight mice (4 male, 4 female) were fed diets with or without added potassium sulphate in the control (low Se) or Se supplemented diets (*n* = 64).

### 2.3. Conventional and Germ-Free Experiments

Germ-free (GF) C57Bl/6 mice and matched specific pathogen free mouse (SPF) mice (4 weeks old) were placed on the low Se diet (0.018 ppm Se) or a 0.3 ppm Se enriched diet (inorganic Se, organic Se and nanoelemental Se) for 5 weeks. Diets were irradiated at 50 kGy (Steritech irradiation, Melbourne, Australia). Six mice (3 male, 3 female) were in each Se enriched group (inorganic, organic or nanoelemental Se) in either GF isolators or a SPF mouse unit in the same facility (*n* = 36). Three mice for each different Se diet formulation were placed on the diet with no added Se (*n* = 12).

### 2.4. Wieghing and Harvesting Mice

Animals were weighed every day for the first 5 days then at longer intervals (weighed on days 1, 2, 3, 4, 5, 8, 11, 15, 22, 29, 36 and day of euthanasia = 37). For harvesting tissues, mice were anesthetised with a mixture of xylazine (20 mg/kg; Troy Laboratories, Sydney, Australia) and ketamine hydrochloride (100 mg/kg; Troy Laboratories) diluted in sterile PBS, given subcutaneously. Terminal blood was collected from the anesthetized mice by cardiac puncture and the mice were then killed by decapitation for collection of tissue. Ileum, liver, skeletal muscle and fecal samples were taken for Se and enzyme determination. Wet weight was recorded and samples were placed in an Eppendorf tube and snap frozen in liquid nitrogen for Se determination by mass spectrometry; ileum and liver were snap frozen for determination of glutathione peroxidase (GPx) protein levels by Western blot, and GPx activity by functional assay. The terminal blood was collected in microvette EDTA tubes (Sarstedt, Mawson lakes, Australia) and spun down (850 g for 10 min at 4 °C) and plasma was collected. The blood plasma was aliquotted into samples for determining GPx activity, Western blot and Se concentrations. Animals were weighed every day for the first 5 days then at longer intervals (weighed on days 1, 2, 3, 4, 5, 8, 11, 15, 22, 29, 36 and day of euthanasia = 37). Weight gains were not different between groups, consistent with other studies [23,24].

### 2.5. Protein Extraction, Glutathione Peroxidase Activity, Western Blotting

Snap frozen liver and ileum samples were sonicated in 5 mL ice cold buffer (50 mM Tris-HCl, 5 mM EDTA, 1 mM DTT, pH 7.5) per 1 g of tissue, followed by centrifugation at 10,000× *g* for 15 min at 4 °C to extract protein. Total protein concentration of the liver and ileum lysates, as well as the plasma samples, were assayed with the Quick Start Bradford Protein Assay Kit (BioRad, Sydney, Australia).

To measure GPx activity, lysates were diluted to the following protein concentrations, in order to fall within the dynamic range of the assay, liver, 0.3 mg/mL; ileum, 3.5 mg/mL and plasma, 10 mg/mL. Extracts were assayed in duplicate using the Glutathione Peroxidase Assay Kit (Cayman Chemical). Bovine erythrocyte GPx was used as a positive control for the assay (see manufacturer’s datasheet). Results are expressed as nMol/min GPx activity per mg total protein (nMol/min/mg).

For Western blot analysis, the same liver and ileum lysates, as well as the plasma samples were diluted to 1 μg/μL in Laemmli sample buffer (BioRad) with 50 mM DTT and boiled for 10 min. Then, 20 μL of each was then loaded onto 4–15% Mini-PROTEAN TGX Stain-Free Protein Gels (BioRad) and electrophoresed at 100 V with 25 mM Tris, 192 mM glycine, 0.1% SDS, pH 8.3 running buffer. Precision Plus Protein All Blue Prestained Protein Standard (BioRad) was used for molecular weight determination. Gels were then placed in a ChemiDoc MP imaging system (BioRad) and exposed to UV light. Gels were then transferred to 0.2 μm nitrocellulose membranes using the Trans-Blot Turbo semi-dry transfer system and transfer packs (BioRad). The Stain-Free total protein labelling on the blot was then imaged, followed by air drying for 1 h, then blocking for 1 h in Odyssey blocking solution (Li-Cor). Primary antibodies (see Table 2) were diluted in Odyssey blocking solution with 0.2% Tween 20 and applied to blots overnight at 4 °C. Blots were washed 3 × 5 min in PBS with 0.1% Tween 20. Secondary antibodies (see Table 1) were diluted in Odyssey blocking solution with 0.2% Tween 20 and applied to blots for 1 h at room temperature. Blots were washed 3 × 5 min in PBS with 0.1% Tween 20. Blots were then imaged on the ChemiDoc (BioRad) and image analysis performed using the ImageLab software (BioRad). Results are expressed as band intensity normalised against total protein intensity, relative to a loading control sample that was run on each gel (pooled from 3 control animals).

### 2.6. Statistics

All data are presented as mean ± SEM. One-way ANOVAs were performed on the datasets with a post hoc Tukey’s multiple comparisons test. We compared between Se groups in bioavailability experiments using a general ANOVA with a post hoc Tukey’s test (See supplement S2 for Mass spectrophotometry and GPx activity. Non-parametric unpaired *t*-tests comparing ranks (Mann–Whitney test) were performed for comparison of ceca weights between GF and SPF mice. Statistical significance is designated as *p* < 0.05 *, *p* < 0.01 **, *p* < 0.001 ***, *p* < 0.0001 ****.

## 3. Results

### 3.1. Levels of Selenium in Mice: Bioavailability Experiments

**Figure 1:**
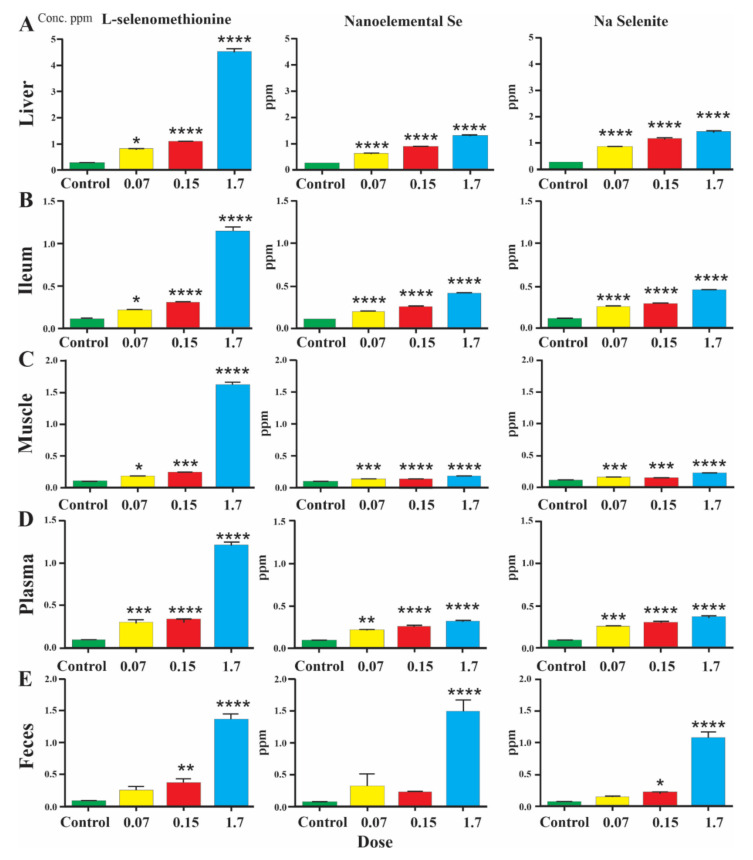
Comparisons of Se distributions in liver (**A**), ileum (**B**), muscle (**C**), plasma (**D**) and feces (**E**) after 5 weeks on control or Se enriched diets, plotted as concentration (parts per million, ppm) measured by mass spectrometry. Distribution of Se in liver, ileum, muscle, plasma and feces were compared relative to the control fed group of animals, i.e., minimal Se diet (0.018 ppm). One-way ANOVA was carried out with Tukey’s multiple comparisons between groups. Data is presented as mean ± SEM. *p* < 0.05 *, *p* < 0.01 **, *p* < 0.001 ***, *p* < 0.0001 ****, relative to control.

**Figure 2:**
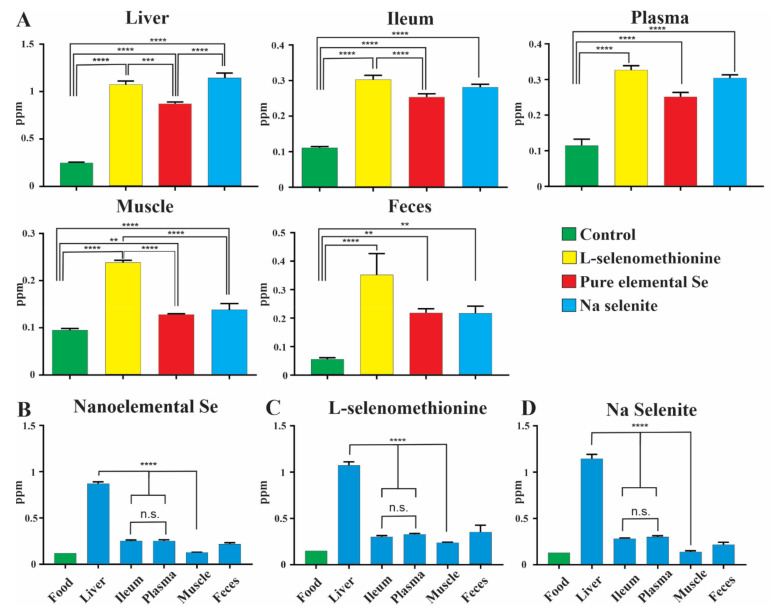
(**A**): Comparison of levels of Se in liver, ileum, plasma, muscle and feces when fed different Se diet formulations for 5 weeks at 0.15 parts per million (ppm) measured by mass spectrophotometry. (**B**–**D**): Comparison of Se concentrations between liver, ileum, plasma, muscle and feces in animals fed a nanoelemental Se, L-selenomethionine and Na Selenite diet at 0.15 ppm for 5 week (*n* = 6 animals for tissues and feces; food, *n* = 1). Statistically significant increases in bioavailability of Se are observed in all diet formulations. One-way ANOVA was carried out with Tukey’s multiple comparisons between groups. Data is presented as mean ± SEM, n.s.: not statistically significant, *p* < 0.01 **, *p* < 0.001 ***, *p* < 0.0001 ****.

**Figure 3:**
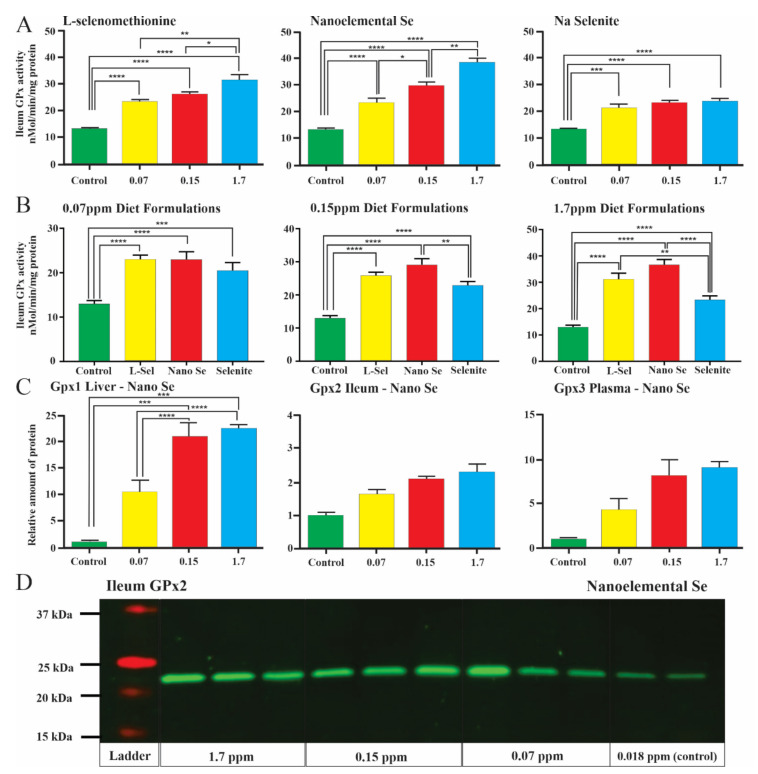
(**A**): glutathione peroxidase (GPx) activity in the ileum after feeding mice with diets containing Se derived from different formulations. (**B**): Comparison of GPx activity in ileum between different Se formulations at a similar dietary level. (**C**): Relative amounts of GPx protein in the liver, ileum and plasma after feeding mice with nanoelemental Se for 5 weeks, as measured by fluorescence intensity, relative to control diets. (**D**): Representative fluorescent immunoblot of GPx2 protein in ileum tissue from mice fed a nanoelemental Se supplemented diet at 0.3, 0.15, 1.7 ppm and control diet (0.018 ppm) for 5 weeks, each lane is an individual protein sample. The dominant GPx form in each tissue was measured. Please note different vertical scales. One-way ANOVA was carried out with Tukey’s multiple comparisons between groups. Data is presented as mean ± SEM. *p* < 0.05 *, *p* < 0.01 **, *p* < 0.001 ***, *p* < 0.0001 ****.

Levels of selenium as measured by mass spectrometry were significantly increased in the liver, ileum, muscle, plasma and feces of mice after 5 weeks on Se enriched diets (Figure 1A–E; note the different scales in the sections of this figure, and Supplementary Table S1). The high Se diet (1.7 ppm) resulted in the highest Se levels in all tissues and plasma, independent on the Se source (Figure 1A–E). Interestingly, despite being ~11 to ~25 times less concentrated, the lower Se diets resulted in comparably high Se levels in liver, ileum and plasma for all Se sources (shown for 0.15 ppm in Figure 2A). With moderate doses of Se (concentrations that could be used in livestock feed), levels in plasma were not significantly different for each of the three formulations (Figure 2A), indicating a similar uptake into the blood stream. Furthermore, liver and ileum Se concentrations were significantly increased in mice fed the nanoelemental Se diet compared to the low Se group, indicating its effective absorption from the diet. The concentrations in liver were statistically significantly greater than in plasma for all levels of the nanoelemental Se diet, indicating hepatic accumulation of Se (Figure 2B). Hepatic accumulation of Se, relative to plasma, was also observed for other Se diet formulations and concentrations. By contrast, Se levels in ileum were similar to those in plasma for all dietary levels and formulations (Figure 1B,D), suggesting Se is effectively bioavailable but does not become concentrated in gut to the degree that it does in liver (Figure 2B). Interestingly, levels of Se in muscle were less than in intestine or plasma, and substantially less than in liver at concentrations of 0.15ppm SeMet Figure 2B–D). Significantly lower levels of Se were found in muscle after inorganic and elemental Se feeding at 0.15 ppm relative to SeMet (Figure 2A), for nanoelemental Se this was about 10% of the liver value and half the intestine value (Figure 2B and Figure 3). Weight gains were not different between groups, consistent with other studies [23,24].

The levels of Se present in the feces with the three formulations were similar, although at a moderate dose in the diet more fecal loss was seen in animals fed organic Se (Figure 1E and Figure 2A). Plasma levels achieved with the 0.07 and 0.15 ppm doses were similar across formulations (0.15 ppm shown in Figure 2A), but with the 1.7 ppm dose were significantly greater for SeMet compared to other Se formulations (Figure 1D). This suggests that the mechanism of incorporation of Se from the inorganic or nanoelemental Se sources are saturated at 1.7 ppm in the diet.

Comparison of Se levels in food, feces, blood plasma and tissue after nanoelemental Se (0.15 ppm) is provided in Figure 2B–D. The Se levels in ileum and plasma were similar, suggesting that Se derived from nanoelemental Se is efficiently absorbed from the diet. However, it must be considered that the ileum values can reflect accumulation over 5 weeks, whereas plasma levels are anticipated to be more labile and reflect accumulation over shorter times. Concentrations in liver were almost four times those in plasma or ileum, reflecting liver accumulation of Se, but muscle levels were low (Figure 2B–D).

### 3.2. Induction of Glutathione Peroxidase Activity

GPx enzyme activity was significantly increased in the ileum at all doses in all Se formulations (Figure 3A,B; Supplementary Table S2). GPx activity in liver and plasma was also increased in all three Se supplemented diets (see Supplementary Figure S1). It is notable that GPx activity in the ileum was as great or greater for nanoelemental Se compared to SeMet or selenite at all three doses (Figure 3B). There was not a strong dose–response relationship (Figure 3A). Although significantly different, doubling the Se dose from 0.07 to 0.15 ppm increased GPx activity by only 5–25%, and a more than 10-fold further increase (to 1.7 ppm) caused only up to 20% increase **(**Figure 3A). These data could suggest that induction of GPx activity is saturable, but this requires further investigation, particularly noting that Se can enter other pathways in addition to incorporation into GPx.

### 3.3. Changes in Enzyme Levels

Protein was extracted from organs and plasma and measured by densitometry of Western blots (Figure 3C). The dominant forms in liver (GPx1), ileum (GPx2) and plasma (GPx3) were each assessed after added dietary Se in each formulation (data for nanoelemental Se is in Figure 3C). Even at the lowest dose, 0.07 ppm, there was an increase in the amount of enzyme protein in liver (Figure 3C). Statistically significant increases in GPx protein levels were difficult to determine in ileum and plasma because protein was pooled for control animals (Figure 3C).

### 3.4. Effect of a Sulphate Free Diet

**Figure 4:**
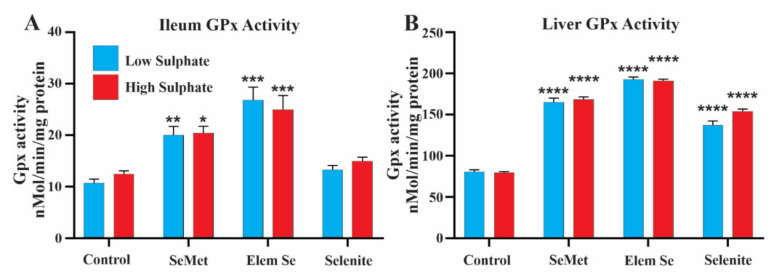
Comparison of GPx activity in low sulphate and high sulphate diets. (**A**,**B**): GPx activity in ileum and liver tissue of mice fed low or high sulphate l-selenomethionine (SeMet), nanoelemental Se or sodium selenite diets at 0.3 parts per million (ppm) for 5 weeks, respectively. One-way ANOVA was carried out with Tukey’s multiple comparisons between groups. Data is presented as mean ± SEM. *p* < 0.05 *, *p* < 0.01 **, *p* < 0.001 ***, *p* < 0.0001 ****. Low or high sulphate diets were compared between Se supplemented and control diets, i.e., low sulphate control compared with low sulphate SeMet.

It is feasible that sulphate handling bacteria could contribute to the conversion of elemental Se to a reduced metabolite in the lumen that could be absorbed and contribute to the formation of selenocysteine and production of selenoprotein (see Background). However, we found that the levels of GPx activity between mice on a nominally sulphate free diet and a sulphate enriched diet (S as K_2_SO_4_, 666 ppm) were not significantly different **(**Figure 4A,B). Consistent with the first trial, the GPx activity in ileum and liver was increased in all Se diet formulations relative to the control fed group except for ileum GPx activity in the selenite supplemented group (Figure 4 and Figure 3B).

### 3.5. Selenium Conversion in Germ-Free (GF) Mice

**Figure 5:**
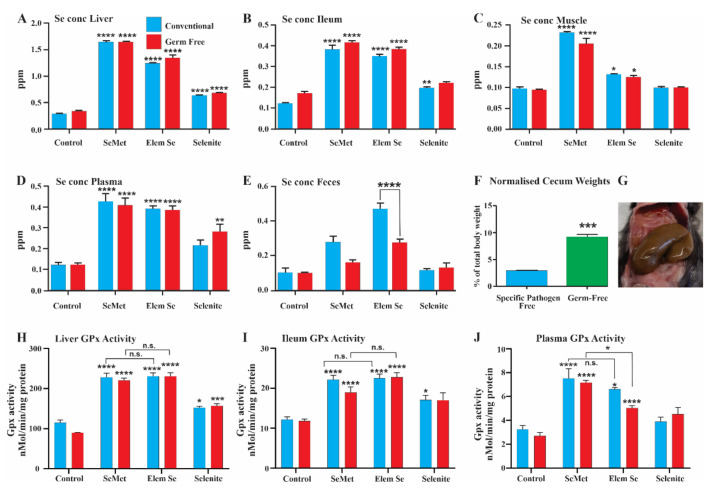
(**A**–**E**): Selenium concentrations in liver, ileum, muscle, plasma and feces of mice after supplementing feed of conventional and germ-free mice with 0.3 ppm organic Se (SeMet), inorganic Se (Selenite) or nanoelemental Se (Elem Se). Se measured by mass spectrometry. (**H**–**J**): GPx activity in the liver, ileum and plasma after feeding mice with diets containing 0.3 ppm Se derived from different formulations; organic, inorganic or nanoelemental Se. (**F**,**G**): Cecum weights. (**F**) shows the difference in cecum weights between SPF and GF mice, relative to overall body weight. Non-parametric unpaired t-test comparing ranks (Mann–Whitney test), *p* < 0.0001. (**G**) shows a photograph of enlarged cecum of a GF animal. (**A**–**E**,**H**–**J**). One-way ANOVA was carried out with Tuckey’s multiple comparisons between groups, statistical significance was measured relative to control fed mice in GF and SPF groups. Data is presented as mean ± SEM. *p* < 0.05 *, *p* < 0.01 **, *p* < 0.001 ***, *p* < 0.0001 ****.

GF and matched SPF mice were fed for 5 weeks on the low Se and Se supplemented diets (0.3 ppm). The ceca of the GF mice were significantly enlarged (Figure 5F,G), which is caused by the movement of body fluid into the cecum, a characteristic feature of GF rodents [25].

At the end of 5 weeks, there was no significant difference between GF and SPF mice in the total Se concentrations in liver, ileum, muscle or plasma measured by mass spectrometry (Figure 5A–E. please note different vertical scales). The increase in fecal Se that occurred with Se supplementation was significantly less (*p* < 0.001) in GF than in SPF mice for the nanoelemental Se diets (Figure 5E). This suggests that bacteria are involved in retention of Se of elemental origin in the feces. Consistent with the first trial, the bioavailability of Se was significantly increased in liver, ileum, muscle and plasma in all Se supplemented diets relative to control, except for Se levels in muscle of selenite supplemented diets (Figure 5A–C).

Consistent with the similar incorporation of Se into the liver and ileum in the first trial, GPx enzyme activity was significantly increased in GF and SPF mice (Figure 5H–J) with each of the formulations (0.3 ppm in the diet). Notably, the level of increased GPx activity induced by dietary nanoelemental Se was quite similar to the increase with organic Se (SeMet) in liver, ileum and plasma tissue (Figure 5H–J); inorganic Se was less effective. This difference in functional activity between the moderate doses of nanoelemental and SeMet groups relative to inorganic Se fed animals was also observed in the first trial (Figure 3B).

## 4. Discussion

**Figure 6:**
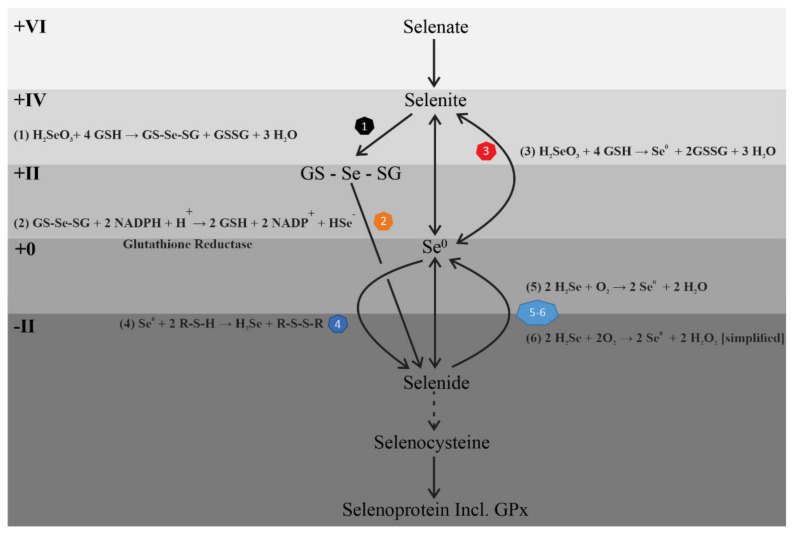
Pathways for formation of selenide, necessary for selenocysteine (SeCys) production, from elemental Se or selenite, and eventual incorporation into selenoproteins, including GPx. Se valencies are indicated at the left. Reaction numbers correspond to those in the text. See details in the text.

The current study revealed that, independent of whether dietary Se is delivered in organic, inorganic or nanoelemental forms, low and moderate amounts of added Se (0.07 to 0.3 ppm in the diet) lead to incorporation of Se into ileum, liver and muscle (Figure 1 and Figure 5A–E). Consistent with previous studies, saturation of GPx activity was observed with all forms of dietary selenium (Figure 3A) [26]. Moreover, nanoelemental Se increased GPx protein and enzyme activity to levels very similar to those caused by organic Se (SeMet) (Figure 3, Figure 4 and Figure 5). The elevation of enzyme activity was greater than with the same Se dose in inorganic form (selenite) at 0.15 and 1.7 ppm (Figure 3B). Despite these differences, that are discussed below, there is a very effective incorporation and reduction in zero-valent nanoelemental Se into bioactive, reduced (Se^−Ⅱ^) selenoproteins. This could feasibly be through bacterial conversion [27]. We tested the influence of sulphate reducing bacteria, which are present in C57/Bl6 mice, on nanoelemental Se incorporation in particular or, more generally, the possible role of other bacterial strains in the intestinal microbiota, using GF mice [28]. These experiments showed that incorporation of Se from the nanoelemental Se source, and induction of GPx enzyme activity, were not dependent on intestinal bacteria (Figure 5). Both lines of evidence therefore point towards an initial reaction of elemental Se with mouse derived metabolites (either intracellularly or in the gastrointestinal tract lumen). Both Se^0^ and Se^+IV^ were incorporated into selenoproteins (Se^−Ⅱ^) with comparable high efficiencies, suggesting a common metabolic pathway (Figure 6).

Selenite and elemental Se both need to be reduced to Se^−Ⅱ^ in order to incorporate selenite Se^+ IV^ or elemental Se (Se^0^) into proteins (Figure 6), via the common intermediate metabolite, H_2_Se (Se^−Ⅱ^) [6,29]. In contrast, SeMet already contains Se in a reduced state (Se^−Ⅱ^) that can be incorporated into selenocysteine by trans-selenation [9]. Selenite is known to react with biogenic thiols such as glutathione (GSH) via seleno (^+II^) diglutathione (reaction 1). Selenodiglutathione (GS-Se-SG) is a highly efficient oxidant of reduced thiols, such as thioredoxin, and is a substrate for mammalian thioredoxin reductase. The overall reaction yielding H_2_Se is depicted below [30,31] (reaction 2):

SeO_3_^2−^ + 4 GSH + 2H^+^ → GS-Se-SG + GSSG + 3 H_2_O (1)

GS-Se-SG + 2 NADPH + H^+^ → 2 GSH + 2 NADP^+^ + HSe^−^ (2)

Interestingly, in the presence of excess reduced glutathione, selenite can also undergo an incomplete reduction (in contrast to reactions 1 and 2) to elemental Se as follows:

H_2_SeO_3_ + 4 GSH → Se^0^ + 2GSSG + 3 H_2_O (3)

(modified from Nuttall, 1984) [31].

This reaction represents a possible pathway linking selenite to elemental Se that could be further reduced to selenide (Figure 6). Se^0^ is known to readily react with numerous sulphur compounds of varying oxidation states, ranging from oxidized, e.g., sulphite (S ^+IV^), through more reduced thiosulfate (S^+Ⅱ^) forms to the most reduced S compounds (e.g., sulfides/thiols S^−Ⅱ^). The generalized thiol reaction yields selenium in fully reduced form (H_2_Se):

Se^0^ + 2 R-S-H →H_2_Se + R-S-S-R (4)

In mammals, redox homeostasis is maintained by corresponding reductases. The oxidized disulfide form of glutathione (GSSG) for instance, is reduced, with NADPH consumption, to GSH by glutathione reductase. Hydrogen selenide is highly reactive (e.g., with chalcophilic co-factors such as Cu, Co, Fe etc) and is prone to oxidation to elemental Se, causing to some degree the generation of reactive oxygen species (simplified) according to:

2 H_2_Se + O_2_ → 2 Se + 2 H_2_O (5)

2 H_2_Se + 2O_2_ → 2 Se + 2 H_2_O_2_ (6)

In addition, generation of reactive oxygen species (ROS) has also been observed upon chemical reaction of GSH and selenite (reaction 1, Figure 6). Nanoelemental Se, like selenite, has been shown to target and rapidly accumulate in cancer cells, producing ROS that leads to cell death [29]. Generation and scavenging of ROS can ultimately explain why with all treatments there was increased GPx protein expression (incorporation of selenocysteine following Se reduction in the cases of sodium selenite or nanoelemental Se to form H_2_Se; or trans-selenation in the case of selenomethionine) and enzymatic activity (eliminating ROS generated) [32].

Our work in vivo implies that after nanoelemental Se enters the small intestine it is reduced, perhaps in multiple steps, and incorporated into selenoproteins, which is consistent with earlier studies demonstrating increases in GPx phospholipid hydroperoxide, and thioredoxin reductase activity in response to dietary nanoelemental Se [24]. If absorption and uptake of nanoelemental Se across the intestinal epithelium occurs in the (unreacted) form, it is likely via endocytotic transcellular transport [33]. The efficiency of this absorption is determined by the size and charge of the nanoparticle [34], which is determined by proteins associated to the Se core (in this formulation, Bovine Serum Albumin) [35]. In agreement with our work, Boostani et al. found increased efficiency of antioxidants in nano-Se in broiler chickens compared to organic and inorganic forms [23]. While selenium is an essential trace element, high levels of intake has toxic effects. However, at higher levels it is notable that nanoelemental Se is less toxic than other forms [33,36], whereas our study suggests it is similarly incorporated at low doses. Elemental Se caused substantial increases in fecal Se of conventional mice, but lesser increases in GF mice (Figure 5E). This difference of Se content in feces could be due to differences in colonic barrier structure of GF compared to conventional mice [37] or, due to an absence of Se accumulating bacteria, as bacteria may compete with the host for dietary Se [38].

An interesting observation was that elemental Se was absorbed as efficiently as selenite, but was closer to selenomethionine in its metabolic effect (increase in GPx activity). This confirms an earlier observation of similarly increased GPx activity in mice fed nanoparticle Se compared to selenomethionine [39]. This is possibly because multiple steps of reduction from selenite to selenide are required, whereas only a single reduction step is required for elemental Se conversion to selenide (Figure 6). A recent study of Se incorporation from a wide range of foods also indicates that Se that is in organic form is more effective than inorganic Se in increasing serum levels of Se [40]. This is consistent with our data that suggests inorganic Se to be less effective than other forms. Although the differences that we observed may be explained in terms of differences in the numbers of steps required to reach selenide, other factors may contribute to the balance of Se forms after 5 weeks of supplementation.

Given its effective uptake, low toxicity [41] and low production cost (our data on production costs are unpublished), nanoelemental Se may be a more viable option for commercial applications than current formulations. This is particularly important considering that there is possibly not sufficient Se available to supply all human and animal needs [42,43]. In fact, many millions of people are already suffering Se deficiency, which may be exacerbated by climate change [43,44]. Moreover, there is a growing competition for Se between nutritional needs and renewable energy technologies such as solar cells [45]. It should also be considered that the daily requirements of Se, and safe levels to administer, continue to be debated [46,47].

## 5. Conclusions

The current study shows that increased GPx protein levels and activity are very similar whether Se is delivered in nanoelemental form or as an organic form. Its absorption and metabolism is not dependent on bacterial conversion.

## Figures and Tables

**Table 1:** Composition of the modified AIN-93G diet sourced from Speciality feeds (low selenium Torula yeast extra vitamins semi-pure rodent diet; SF16-104).

Ingredients	Concentration (g/kg)
Low Selenium Torula Yeast	300
Sucrose	100
Canola oil	70
Cellulose	50
Wheat Starch	302
Dextrinised Starch	132
L Methionine	6.3
L Tryptophan	0.17
Calcium Carbonate	24
Sodium Chloride	1.7
Modified AIN93G Trace Minerals (No added selenium)	1.4
Choline Chloride (75%)	2.5
AIN93 Vitamins	15
Vitamin K	0.87

**Table 2:** Primary and secondary antibodies used for Western blot analysis.

Antibody	Supplier	Dilution Used
Goat Anti-Glutathione Peroxidase 1	ab140883 (abcam Melbourne, Australia)	1:500
Rabbit Anti-Glutathione Peroxidase 2	ab137431 (abcam)	1:3000
Goat Anti-Glutathione Peroxidase 3	AF4199 (R&D Systems, Melbourne, Australia)	1:1500
Donkey anti-Rabbit IgG (H + L) Secondary Antibody, Alexa Fluor^®^ 488 conjugate	A-21206 (Molecular Probes Melbourne, Australia)	1:2000
Donkey anti-Sheep IgG (H + L) Secondary Antibody, Alexa Fluor^®^ 488 conjugate	A-11015 (Molecular Probes)	1:1000

## Data Availability

Data is available by contacting the first named author.

## Supplementary Materials

The following are available online at https://www.mdpi.com/2072-6643/13/4/1073/s1, Figure S1: GPx activity in the liver and plasma, Table S1: Distribution of Se from selenomethionine, nanoelemental Se, and Na Selenite at 0.07, 0.15, and 0.1.7 ppm respectively in mouse tissues and feces. Table S2: GPx activity (nMol/min/mg protein) from selenomethionine, nanoelemental Se, and Na Selenite at 0.07, 0.15, and 0.1.7 ppm respectively in mouse tissues. C refers to concentration and D refers to diet.
