# Supplementary material for: Analysis of Bioavailability and Induction of Glutathione Peroxidase by Dietary Nanoelemental, Organic and Inorganic Selenium
Source: Nutrients. 2021 Mar 25;13(4):1073. doi: 10.3390/nu13041073 (PMC8067071; doi:10.3390/nu13041073)
Supplement: Supplementary file 1 [file nutrients-13-01073-s001.pdf]

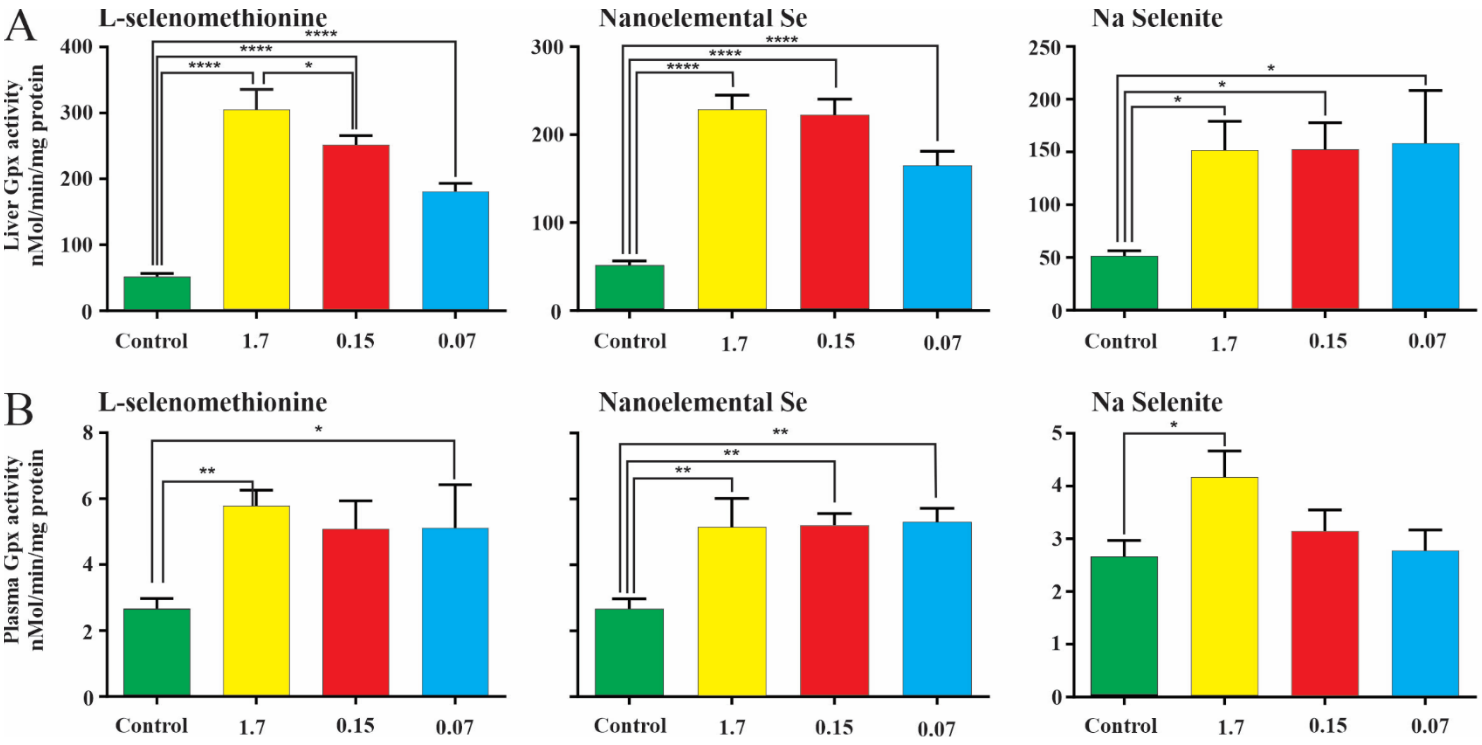

Figure S1: GPx activity in the liver and plasma

Table S1: Distribution of Se from selenomethionine, nanoelemental Se, and Na Selenite at 0.07, 0.15, and 0.1.7 ppm respectively in mouse tissues and feces.

Parameter	Control^{+}	Na Selenite			Selenomethionine			Nanoelemental Se			SED	P-value
	0.0018ppm	0.07ppm	0.15ppm	1.7ppm	0.07ppm	0.15ppm	1.7ppm	0.07ppm	0.15ppm	1.7ppm	D x C	
Liver	0.24	0.835^{abc}	1.15^{d}	1.42^{e}	0.772^{ab}	1.07^{cd}	4.47^{f}	0.587^{a}	0.872^{bc}	1.23^{de}	0.080	<.001
Ileum	0.11	0.238^{ab}	0.282^{ab}	0.430^{c}	0.205^{a}	0.302^{b}	1.13^{d}	0.193^{a}	0.253^{ab}	0.412^{c}	0.030	<.001
Plasma	0.193	0.248^{ab}	0.300^{abc}	0.360^{c}	0.288^{abc}	0.327^{bc}	1.20^{d}	0.215^{a}	0.252^{ab}	0.322^{bc}	0.032	<.001
Muscle	0.001	0.142^{ab}	0.138^{ab}	0.205^{bc}	0.183^{abc}	0.238^{c}	1.62^{d}	0.123^{a}	0.128^{ab}	0.172^{abc}	0.024	<.001
Feces	0.055	0.127^{a}	0.217^{a}	1.05^{b}	0.233^{a}	0.352^{a}	1.35^{bc}	0.319^{a}	0.218^{a}	1.62^{c}	0.160	0.155

[+] *all treatments higher than control. SEM (standard error of mean) for control were 0.009, 0.003, 0.009, 0 and 0.005 for liver, ileum plasma, muscle and feces, respectively). Pooled standard error of the difference (SED). C refers to concentration and D refers to diet.*

Table S2: GPx activity (nMol/min/mg protein) from selenomethionine, nanoelemental Se, and Na Selenite at 0.07, 0.15, and 0.1.7 ppm respectively in mouse tissues. C refers to concentration and D refers to diet.

Parameter	Control[+]	Na Selenite			Selenomethionine			Nanoelemental Se			SED	P-value
	0.0018ppm	0.07ppm	0.15ppm	1.7ppm	0.07ppm	0.15ppm	1.7ppm	0.07ppm	0.15ppm	1.7ppm	D x C	
Liver	51.43[+]	158.3[a]	152.4[a]	151.7[a]	180.7[a]	251.5[ab]	305[b]	164.9[a]	222.2[ab]	220.8[ab]	37.55	0.18
Ileum	12.98[+]	20.5[a]	22.89[ab]	23.44[ab]	23.03[ab]	25.86[abc]	31.24[cd]	22.99[ab]	29.12[bc]	36.68[d]	2.277	0.03
Plasma	2.66	2.78[a]	3.14[a]	4.17[a]	5.11[a]	5.19[a]	5.78[a]	5.29[a]	5.2[a]	5.15[a]	0.953	0.844

[+] *all treatments higher than control. SEM (standard error of mean) for control were 4.93, 0.76 and 0.3 for liver, ileum and plasma, respectively). Pooled standard error of the difference (SED). C refers to concentration and D refers to diet.*
